# NOX4 Regulates CCR2 and CCL2 mRNA Stability in Alcoholic Liver Disease

**DOI:** 10.1038/srep46144

**Published:** 2017-04-06

**Authors:** Yu Sasaki, Ali Dehnad, Sarah Fish, Ai Sato, Joy Jiang, Jijing Tian, Kathrin Schröder, Ralf Brandes, Natalie J. Török

**Affiliations:** 1 Division of Gastroenterology and Hepatology, University of California Davis, Sacramento, CA, USA; 2 VA Northern California Health Care System, Mather, CA, USA; 3 Wolfgang Goethe University, Frankfurt am Main, Germany

## Abstract

Recruitment of inflammatory cells is a major feature of alcoholic liver injury however; the signals and cellular sources regulating this are not well defined. C-C chemokine receptor type 2 (CCR2) is expressed by active hepatic stellate cells (HSC) and is a key monocyte recruitment signal. Activated HSC are also important sources of hydrogen peroxide resulting from the activation of NADPH oxidase 4 (NOX4). As the role of this NOX in early alcoholic liver injury has not been addressed, we studied NOX4-mediated regulation of CCR2/CCL2 mRNA stability. NOX4 mRNA was significantly induced in patients with alcoholic liver injury, and was co-localized with αSMA-expressing activated HSC. We generated HSC-specific NOX4 KO mice and these were pair-fed on alcohol diet. Lipid peroxidation have not changed significantly however, the expression of CCR2, CCL2, Ly6C, TNFα, and IL-6 was significantly reduced in NOX4^HSCKO^ compared to *fl/fl* mice. NOX4 promoter was induced in HSC by acetaldehyde treatment, and NOX4 has significantly increased mRNA half-life of CCR2 and CCL2 in conjunction with Ser221 phosphorylation and cytoplasmic shuttling of HuR. In conclusion, NOX4 is induced in early alcoholic liver injury and regulates CCR2/CCL2 mRNA stability thereby promoting recruitment of inflammatory cells and production of proinflammatory cytokines.

Alcoholic liver disease (ALD) is a major cause of morbidity and mortality in the US and worldwide. The exact pathomechanism is still only partially understood, but severe oxidative injury and recruitment of monocytic cells/macrophages and neutrophils are uniformly present and hallmark disease progression. Following alcohol consumption, reactive oxygen species (ROS) are produced by various sources in the liver such as CYP2E1, mitochondrial respiratory chain, and the nicotinamide adenine dinucleotide phosphate (NADPH) oxidases (NOX)^1^,^2^. Amongst the seven NOX homologues, NOX1, NOX2, and NOX4 are the main sources of ROS in the liver^3^ Several studies have shown the importance of NOX activation in ALD, as mice lacking p47^phox^ (required for the activation of NOX1 and NOX2) develop attenuated ALD^4^. NOX4 a non-phagocytic NOX, is mainly transcriptionally activated and directly produces H_2_O_2_^5^. NOX4 is induced in several fibrosis models including non-alcoholic steatohepatitis (NASH) and contributes to the fibrogenic activation of hepatic stellate cells (HSC). However the role of NOX4 in ALD with or without co-existing fibrosis has not been sufficiently evaluated. Activated HSC are the major fibrogenic cell types in the liver^6^,^7^,^8^,^9^ but they were also shown to have a significant immunomodulatory role by promoting homing and activation of tissue macrophages^10^ via the induction of C-C chemokine receptor type 2 (CCR2) in both the bile duct ligation (BDL) and carbon tetrachloride (CCl4) models of fibrosis^11^. CCL2, (also known as MCP1) was also described as a key mediator of inflammation in chronic ALD^12^, and CCL2 levels correlated with disease severity^13^. Despite these observations, the mechanisms and signals responsible for CCR2/CCL2 induction in HSC and consequent recruitment of monocyte/macrophages have not been described.

Here, we demonstrated that NOX4 is induced in patients and in mice with ALD. The alcohol metabolite, acetaldehyde was implicated in the transcriptional activation of NOX4 in HSC, and downstream in the oxidative dysregulation of the mRNA binding protein human antigen R (HuR) shuttling and subsequent increased stability of the CCR2/CCL2 mRNAs. This, in turn resulted in an increase in pro-inflammatory signals in early alcoholic liver injury. The NOX4/CCR2/CCL2 axis therefore could be a potential treatment target to mitigate recruitment of inflammatory cells and injury in ALD.

## Results

### NOX4 is induced in patients with alcoholic liver disease

**Figure 1:**
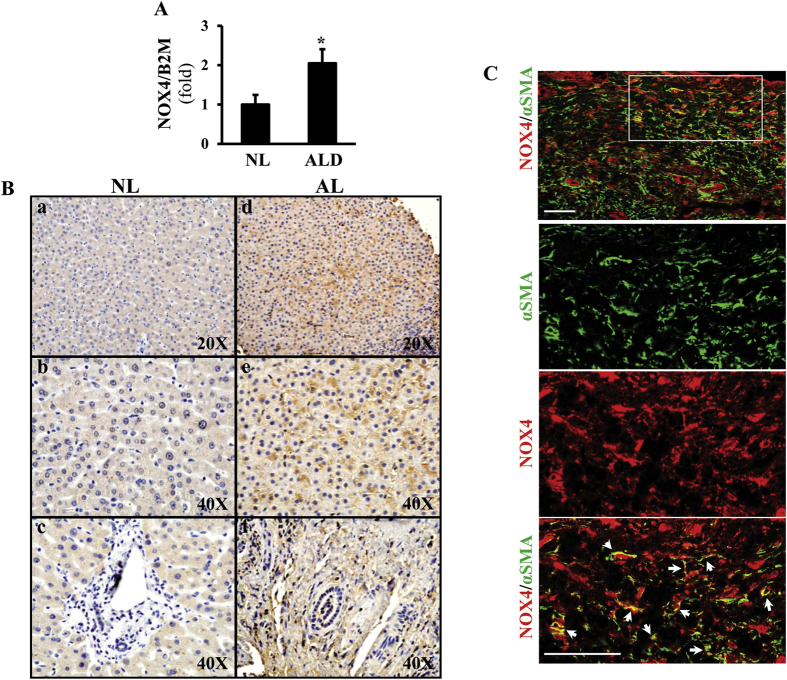
NOX4 is induced in patients with alcoholic liver disease. Liver biopsy samples from healthy individuals (N = 4, NL), and patients with alcoholic liver disease (ALD) (N = 4) were analyzed by RT qPCR for NOX4 expression (**A**). NOX4 was significantly upregulated in ALD (p < 0.05). Immunohistochemistry (IHC) showed increased expression of NOX4 in the biopsy samples of ALD patients both in hepatocytes and the periportal area (d, e, f, (**B**)). Confocal imaging following immunofluorescence staining for NOX4 (red) and αSMA (green) show enlarged area (box) for colocalization of NOX4 and αSMA in liver samples from patients with ALD (arrows, (**C**)). Bar = 100 μm.

To study NOX4 in patients with ALD, we performed real time qPCR (Fig. 1A), immunohistochemistry (Fig. 1B), and immunofluorescence studies to localize NOX4 and αSMA positive activated HSC with confocal microscopy (Fig. 1C). NOX4 was significantly induced in patients with ALD (p < 0.05). Immunohistochemistry demonstrated increased NOX4 signal in hepatocytes as well as in the periportal area in biopsy samples of ALD (Fig. 1B,d–f) while the periportal area in normal livers was devoid of NOX4 signal (Fig. 1B,c). To further delineate the histological localization of NOX4, confocal imaging was performed after immunofluorescence studies. These showed colocalization of NOX4 and αSMA in activated HSC (Fig. 1C).

### NOX4 ^HSCKO^ mice exhibit attenuated levels of pro-inflammatory cytokines/chemokines after alcohol intake

**Figure 2:**
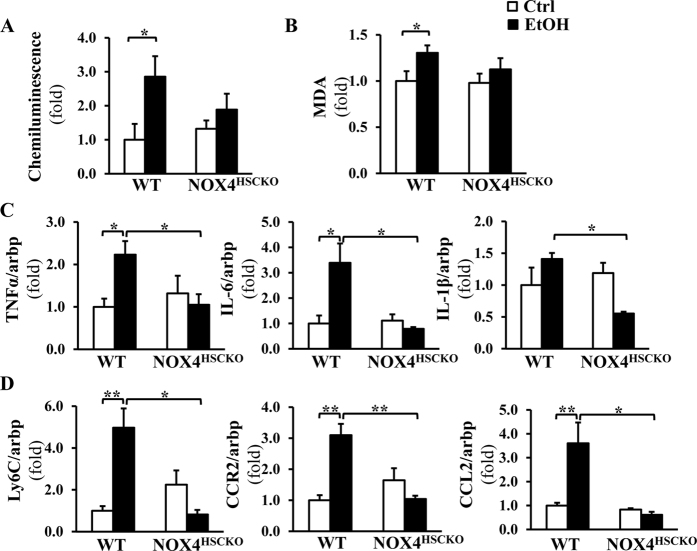
CCR2/CCL2 and inflammatory cytokines are attenuated in the NOX4^HSCKO^ mice on ethanol diet. ROS production and lipid peroxidation were measured by the lucigenin chemiluminescence assay (**A**) and MDA quantitative assays, respectively (**B**). Chemiluminescence results and MDA concentrations were normalized for protein concentrations and the results show increased ROS production and lipid peroxidation in WT mice on alcohol diet, but no significant change in NOX4^^HSCKO^^mice (a trend towards less ROS and MDA, albeit not statistically significant). The expression of pro-inflammatory cytokines TNF-α, IL-1β, and IL-6 (**C**), Ly6C, CCR2, and CCL2 (**D**) were studied by RT-qPCR, and were significantly increased in WT mice on ethanol diet (TNFα (2.4 ± 0.3), IL-6 (2.8 ± 0.3), CCL2 (2.2 ± 0.5), IL-1β (1.4 ± 0.1), Ly6C (4.9 ± 0.9) and CCR2 (3.1 ± 0.3), whereas no induction was observed in NOX4^^HSCKO^^mice (Mean ± SEM, N = 3–4, *p < 0.05, **p < 0.01).

To investigate the potential role of NOX4 in activated HSC modulating pro-inflammatory signaling, NOX4^HSCKO^ and control *fl/fl* (henceforth WT) mice were pair-fed with Lieber-DeCarli alcohol liquid diet or isocaloric control diet for 5 weeks. Genotypes of liver cell populations was confirmed after isolating hepatocytes from control and alcohol diet fed mice; and also for non-parenchymal cells after FACS sorting to gain HSC and Kupffer cell populations (Suppl. Fig. 1). The data showed significant induction of ROS production and lipid peroxidation (MDA) in wild type but not in NOX4^HSCKO^ mice on alcohol diet (Fig. 2A,B). Also, steatosis and triglyceride levels were significantly increased in wild type and less (although not significantly) in NOX4^HSCKO^ mice (Suppl. Fig. 2). Therefore next we focused on the expression of pro-inflammatory cytokines TNF-α, IL-1β, and IL-6 and found that they were significantly reduced in the NOX4^HSCKO^ mice (Fig. 2C), despite no significant change in lipid peroxidation. Ly6C and CCL2, markers of infiltrating monocytic cells, were significantly decreased as well, indicating reduced recruitment (Fig. 2D). As binding of the inflammatory chemokine CCL2/MCP-1 to the chemokine receptor CCR2 is a key to effective recruitment and activation of macrophages^14^,^15^, next we focused on CCR2 expression. While CCR2 was significantly induced in WT mice on alcohol diet; in NOX4^HSCKO^ mice no increase was seen, suggesting that NOX4/ROS in activated HSC regulate CCR2 (Fig. 2D).

### Acetaldehyde induces CCR2 in mouse primary HSC via NOX4

**Figure 3:**
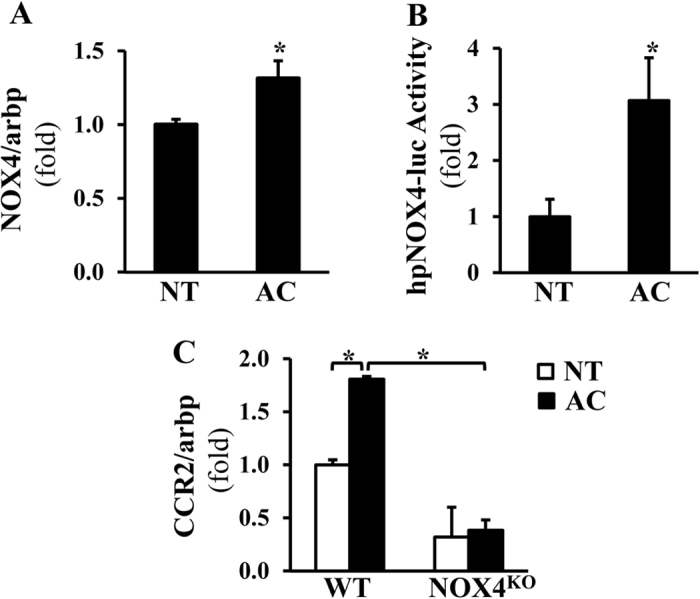
CCR2 induction by acetaldehyde is mediated by NOX4 in hepatic stellate cells. Real time-qPCR shows NOX4 induction following acetaldehyde (AC, 100 μM, 24 hours) treatment in primary HSC (**A**, Mean ± SEM, *p < 0.05). LX-2 cells were transfected with the human NOX4 Luc reporter construct or control construct followed by AC treatment. The reporter assays indicate significantly induced NOX4 promoter activation in AC-treated cells (**B**, Mean ± SEM, *p < 0.05). Primary HSC from WT and NOX4^KO^ mice were isolated and treated with 100 μM AC for 24 hours, and the mRNA levels of CCR2 were assessed by RT-qPCR. Acetaldehyde significantly increased CCR2 mRNA in WT, but not in NOX4^KO^ cells. (**C**, Mean ± SEM, *p < 0.05).

To further investigate the mechanism by which alcohol diet-induced NOX4/ROS dysregulate CCR2 in HSC, we first studied NOX4 expression after acetaldehyde exposure. As HSC express very low levels of alcohol dehydrogenase, we exposed cells to the metabolite acetaldehyde, and found the NOX4 mRNA levels significantly increased (of note, NOX4 is mainly transcriptionally activated, Fig. 3A, p < 0.05). To further provide direct evidence of NOX4 transcriptional activation by acetaldehyde, luciferase reporter assays were performed after transfecting human LX-2 stellate cells with the human NOX4 promoter or control constructs. After 24 hours of acetaldehyde treatment, the NOX4 promoter was significantly induced (Fig. 3B, p < 0.05). To show that NOX4 is required for acetaldehyde-mediated CCR2 induction, primary HSC from WT and NOX4^KO^ mice were isolated. Acetaldehyde treatment resulted in significant increase of CCR2 mRNA levels in *fl/fl* (WT) cells however no increase was seen in NOX4^KO^ cells (Fig. 3C, p < 0.05). We have also tested whether CCR2/CCL2 from HSC is able to induce macrophage migration using a Transwell system (Suppl. Fig. 3). Here we confirmed that induction of CCR2/CCL2 even in NOX4KO HSC is able to induce macrophage migration.

### NOX4 activation results in increasing CCR2 and CCL2 mRNA stability in LX-2 cells

**Figure 4:**
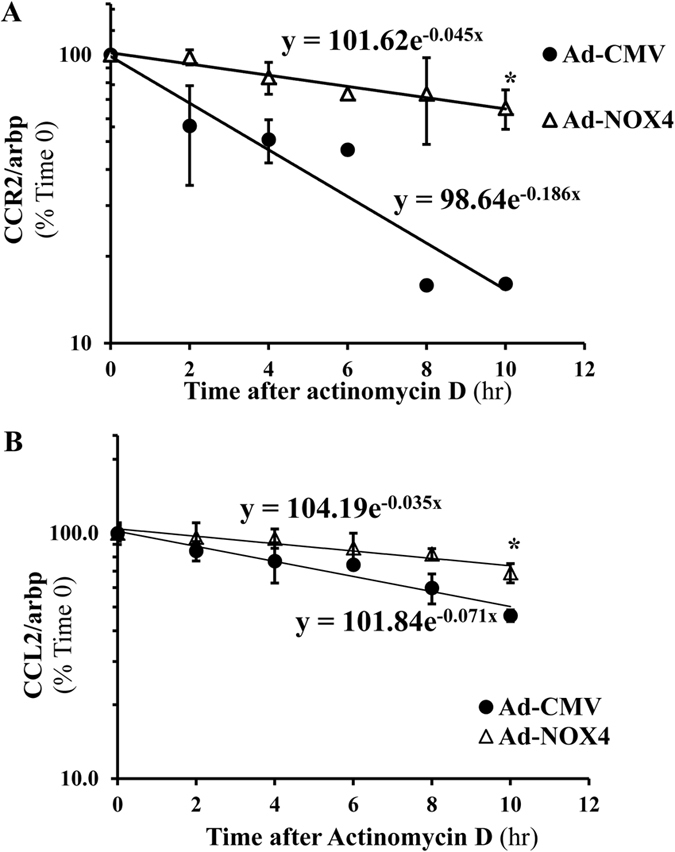
NOX4 stabilizes CCR2 and CCL2 mRNAs in stellate cells. 24 hours after transducing LX-2 cells with adeno-NOX4 or control vector (adeno-CMV), cells were treated with actinomycin D (2 μg/ml) to inhibit RNA polymerase. CCR2 and CCL2 mRNA half-lives were calculated after 2, 4, 6, 8 and 10 hours of treatment as T1/2 = Ln(2)/λ. The result showed significantly prolonged CCR2 (**A**) and CCL2 (**B**) mRNA half-lives in cells transduced by adeno-NOX4 (compared to control vector) *p < 0.05.

LX-2 human stellate cell line has been shown to have all the key features of activated HSC and has been used in numerous studies in hepatic fibrogenesis^16^,^17^, and it expresses NOX4 (Suppl. Fig. 4). As CCR2/CCL2 were downstream from NOX4, next we studied whether induction of NOX4 in HSC results in prolonged CCR2 and CCL2 mRNA stability. Steady-state levels of mRNA are regulated by multiple pathways including transcriptional, turnover and degradation events^18^. As mRNA stability was shown to be affected by oxidative regulation, LX-2 cells were transduced with adeno-NOX4 or control viral vector (ad-CMV) for 24 hours, followed by treatment with actinomycin D (2 μg/ml) to inhibit RNA polymerase and block transcription. CCR2 and CCL2 mRNA half-lives were calculated after 2, 4, 6, 8 and 10 hours. The results indicated that NOX4 induction in LX-2 cells resulted in a significantly prolonged CCR2 and CCL2 mRNA half-lives and therefore more stable mRNAs (Fig. 4).

### Acetaldehyde mediates HuR shuttling from nucleus to cytoplasm via NOX4, and HuR regulates mRNA stability of CCR2 and CCL2

**Figure 5:**
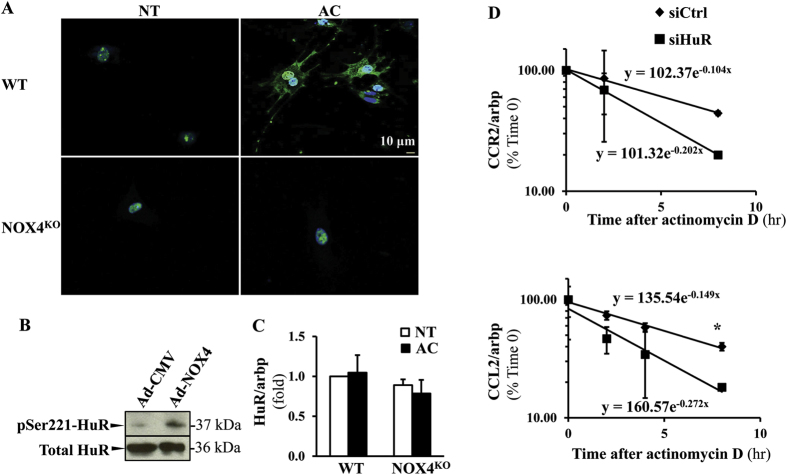
HuR shuttling to the cytoplasm is induced by NOX4 activation in HSC in response to acetaldehyde, and HuR controls CCR2 and CCL2 mRNA stability. Primary HSC from WT and NOX4^KO^ mice were isolated and treated with 100 μM AC for 24 hours. Immunofluorescence studies using HuR antibody (green) were performed with results showing that in response to AC treatment HuR is translocated to the cytoplasm in WT HSC but not in NOX4KO cells. (**A** Blue: DAPI, nucleus. Scale: 10 μm). LX-2 cells were transduced by adeno-NOX4, or control vector. Western blot analysis showed phosphorylation of HuR at S221 in response to NOX4 activation in the cytoplasmic extracts of the cells transduced by Ad-NOX4 (**B**). mRNA levels of HuR did not change following NOX4 transduction (**C**). To address the role of HuR in regulating CCR2/CCL2 mRNA stabilities, LX-2 cells were transfected by control siRNA or siHuR, and 24 hours later cells were treated with actinomycin D (2 μg/ml) to inhibit RNA polymerase. CCR2 and CCL2 mRNA half-lives were calculated after 2, 4, and 8 hours of treatment as T1/2 = Ln(2)/λ. Both CCR2/CCL2 were regulate by HuR (**D**, p < 0.05).

HuR mRNA binding protein was shown to be phosphorylated at S221 by protein kinase C in response to oxidative stress, resulting in its translocation to the cytoplasm, and stabilization of target mRNAs^19^,^20^. HuR is expressed in activated HSC, and HuR silencing attenuated oxidative stress and hepatic fibrosis in the BDL model^21^. To show the effects of acetaldehyde on HuR shuttling, we treated the primary HSC from *fl/fl* (wt) and NOX4^KO^ mice with acetaldehyde. We found that HuR translocated from the nucleus to the cytoplasm in wt but not in NOX4^KO^ HSC (Fig. 5A). In addition, when compared to control vector-transduced HSC, adeno-NOX4 induced the phosphorylation of HuR (S221) as detected in the cytoplasmic fraction (Fig. 5B, full length gel is included in the Suppl. files). HuR mRNA expression as assessed by real-time qPCR however, was not affected in Ad-NOX4-transfected cells (Fig. 5C). To study if HuR is indeed a key to regulate CCR2/CCL2 mRNA stability in HSC, LX-2 cells were transfected with scrambled siRNA or siHuR, and mRNA stabilities were assessed as described above. In cells transfected by siHuR, CCR2/CCL2 mRNA stabilities have decreased significantly (Fig. 5D).

## Discussion

Myofibroblasts play a critical role in fibrogenesis and are the key sources of ECM production. HSC are activated by different mechanisms^22^, and generation of oxidative radicals as a result of NOX activation is an important initiating pathway of HSC activation^5^. NOXs - particularly NOX2 and 4 - regulate collagen I promoter activity^23^,^24^, and ROS also plays a role in maintaining survival signals^25^ in activated HSC. Beside their fibrogenic activity, myofibroblasts can also modulate innate immunity during alcoholic liver injury^26^. According to current understanding, this occurs either indirectly by regulating Kupffer cell activity *via* intricate cross-talks^27^,^28^, or by playing a role in NK cell activation *via* e.g. RAE-1^29^. Recruitment of inflammatory cells to the liver is a key component of disease progression. During alcoholic injury chemokines of the CC and CXC families play a role in the homing of monocytes, neutrophils, T and B lymphocytes. CCL2 was shown to have a particular significance as its increased levels were significantly correlated with IL-8 levels in patients with ALD^13^. CCR2 is the functional receptor for CCL2^30^,^31^, and CCR2-CCL2 binding critically promotes Ly6c+/Gr1 (high) monocyte recruitment to the liver from the bone marrow^32^. The mechanisms of CCR2 and CCL2 induction however are not well understood in alcoholic injury, especially in the milieu of increased oxidative stress. As CCR2 in HSC was shown to be important in fibrosis^11^, and in the recruitment of macrophages in NASH^33^,^34^, we focused on delineating the link between NOX4 activation, production of H_2_O_2_ and activation of CCR2/CCL2. NOX4 is a non-phagocytic member of the NOX family of enzymes, and it is distinct from the other homologues as it is mainly transcriptionally regulated producing H_2_O_2_. Our group and others have shown NOX4 induction in human myofibroblasts^36^, and Suppl. Fig. 4; in addition we earlier demonstrated that NOX4 mediated signals play a role in NASH by dysregulating ER stress pathways and promoting fibrosis. Inhibition of NOX1/4 ameliorated NASH^35^ and CCl4-mediated liver injury^36^. Since the role of NOX4 in alcoholic injury has not been investigated; furthermore as this NOX homologue is a potential treatment target with a potent inhibitor, our goal was to address its role in maintaining an upregulated CCR2/CCL2 cascade. First, we found that NOX4 was induced in patients with alcoholic liver injury both in HSC and hepatocytes. While NOX4 induction in hepatocytes could significantly contribute to alcoholic liver injury, and this can be further studied in the future, here we focused on HSC-mediated pathways as these cells are important sources of CCR2. We have generated a HSC specific NOX4 KO model to further study the role of downstream signaling. The GFAP-cre-based models were criticized in the past as non-specific, in our studies we have not found significant deletion from hepatocytes and Kupffer cells do not have significant NOX4 expression at baseline or during injury. The NOX4^HSC KO^ mice had attenuated proinflammatory cytokines, whereas lipid peroxidation has not decreased as NOX4 was intact in hepatocytes. To address the potential mechanism, we found significant downregulation of both CCR2 and its ligand CCL2 furthermore, Ly6C marker of bone marrow derived macrophages in NOX4^HSCKO^ mice. While the Lieber-deCarli pair feeding model that we used is not a robust model for fibrosis, it can detect early changes due to oxidative stress and recruitment of inflammatory cells^12^,^37^,^38^. To further address the mechanism of NOX4-mediated CCR2/CCL2 induction, we studied if their mRNA stability could be altered by oxidative mechanism. Exposure to oxidative radicals could modify the half-life of mRNAs thereby leading to an increase in their steady-state levels^39^,^40^. Central to this are the RNA binding proteins that by modifying turnover events can affect mRNA half-life^41^. HuR, an mRNA stabilizing factor, is highly expressed in all organs and specifically binds to AU-rich elements of mRNA molecules^42^. HuR is mainly located in the nucleus, but upon various stimuli HuR can be phosphorylated at Ser221 by protein kinase C, and translocate to the cytoplasm^20^. HuR has been shown to play an important role in HSC activation with HuR silencing resulting in an attenuated fibrosis in the BDL and CCl_4_ models of fibrosis^21^. In our experiments transduction of adenoviral NOX4 resulted in increased CCR2 and CCL2 mRNA half-lives. In addition, in transduced cells there was an increase in cytoplasmic HuR (phosphorylated at Ser221). Thus activated HSC *via* an increased production of H_2_O_2_ are able to modulate CCR2/CCL2 mRNA stabilities and thereby the recruitment of monocytic cells from the bone marrow. This effect is likely to be an early event in alcoholic injury preceding frank fibrosis. As CCR2 is also produced by other cells, e.g. the Kupffer cells we cannot rule out that a similar, oxidative stress-mediated mechanism plays a role in CCR2 induction in these cells. While Kupffer cells are not major sources of NOX4, other NOXs e.g. NOX1 or 2 can have similar effects in these cells. We also found an mRNA stabilizing effect of NOX4 on CCL2 mRNA, likely augmenting CCR2/CCL2 signaling in these cells. CCL2 was also shown to have a CCR2 independent effect in alcoholic injury whereby it inhibits PPARα in hepatocytes, contributing to modulation of fatty acid metabolism and steatosis^12^. Of note in our studies we have not observed a significant effect on liver steatosis. NOX4 of course, could also modulate other important chemokines (e.g. CCR9^43^) therefore could act as a proximal regulator of mRNA stability. Also, we cannot exclude the possibility that NOX4 induction in HSC can elicit proinflammatory effects e.g. by differential release of microvesicles.

NOXs have been described as important contributors to oxidative stress in alcoholic liver disease^4^,^44^. P47phox^−/−^ mice were protected from liver injury suggesting that beside NOX4, NOX1 and 2 also may have a significant effect, likely *via* modulating macrophage polarization and/or neutrophil function. It is important to note that we have not found compensatory induction of NOX1 or 2 in our studies (data not shown), although increased enzymatic activity cannot be excluded.

Our study elucidated how the ethanol metabolite acetaldehyde induced NOX4 promoter activity in stellate cells, and this in turn, resulted in increased cytoplasmic shuttling of HuR and CCR2/CCL2 mRNA stability. While activated stellate cells are the main effector cells of fibrosis by production of extracellular matrix, these studies highlight a novel role: modulation of macrophage recruitment in early alcoholic injury. These experiments indicate that interventions to reduce NOX4 or to increase CCR2/CCL2 turnover may attenuate ethanol mediated oxidative stress in HSC at early stages, that later on may result in decreased liver fibrosis.

## Materials and Methods

### Human liver tissues

Liver biopsy FFPE samples were obtained from the UC Davis Cancer Center Biorepository funded by the National Cancer Institute. Samples from 4 different patients with alcoholic liver disease and 4 normal livers were used in the studies. The tissues were processed for immunohistochemistry and real-time qPCR assays.

### Animals and cell culture

All animal experiments were conducted according to the experimental procedures approved by the Institutional Animal Care and Use Committee at the University of California Davis. Conditional HSC NOX4 knockout mice (NOX4^HSCKO^) were generated by crossing the *fl/fl* NOX4 mice with C57B6 background (from R. Brandes, Goethe University, Frankfurt, Germany) and GFAP-cre mice (B6.Cg-Tg(Gfap-cre)73.12Mvs/J, Stock #012886, Jackson Laboratory, Bar Harbor, ME) through several generations. The cell specific knockout of NOX4 was verified with cell isolation and FACS sorting, followed by RT qPCR. In brief, mice were subjected to sequential liver perfusion and enzyme digestion, and hepatocytes were isolated first as described previously^45^. Then the non-parenchymal fraction was labeled with PE-conjugated anti-F4/80 (Biolegend, San Diego, CA) and FACS sorting was performed to purify F4/80+ macrophages and auto-fluorescent HSC. The cells were processed for PCR to analyze NOX4 mRNA. NOX4^HSCKO^ or *fl/fl* NOX4 control littermates were pair-fed with the Lieber-DeCarli alcohol liquid diet (4% vol/vol, BioServe Inc., Flemington, NJ) and isocaloric control for 5 weeks following the manufacturer’s instructions.

Primary stellate cells were isolated from mice as described previously^45^. The cells were cultured in medium 199 (Millipore Sigma, St. Louis, MO) with 20% fetal bovine serum (FBS) and antibiotics. Human immortalized HSC, LX-2 cells were maintained in DMEM supplemented with 5% FBS and antibiotics.

### Immunohistochemistry and immunofluorescence

The FFPE sections from ALD patients and normal controls were deparaffinized and boiled in citric buffer (pH = 6.0) for antigen retrieval. After blocking, the tissues were incubated with anti-NOX4 (1:1000, kindly provided by Dr. A. Shah, King’s College London, UK) at 4 °C overnight. The tissues were reacted with biotinylated secondary antibody followed by diaminobenzidine staining. For immunofluorescence, the slides were incubated with antibodies to NOX4 and αSMA (1:1000; Epitomics, Burlingame, CA) then probed with appropriate fluorescence conjugated secondary antibodies (Life Technologies, Carlsbad, CA). *In vitro*, after 100 μM acetaldehyde treatment for 24 hours, primary HSC were fixed with 4% paraformaldehyde, and then permeabilized with 0.2% TritonX-100 and 0.1% tween 20 in PBS. After blocking, the cells were probed with anti-HuR antibody (1:50, Santa Cruz Biotechnology, Inc. Santa Cruz, CA), followed by appropriate fluorescence secondary antibody (Life Technologies) and mounted with 4′,6-diamidino-2-phenylindole (DAPI). Images were obtained by confocal microscopy.

### Lucigenin assay

ROS production was studied using lucigenin assay. Fresh liver tissues were homogenized on ice in sucrose buffer (0.3 M sucrose, 10 mM HEPES, 10 mM KCl, 0.1 mM EDTA, 0.1 mM EGTA, 0.5 mM Spermidine, 1 mM DTT pH 7.4, with protease inhibitors (PI, Roche, Basel, Switzerland). The homogenate was centrifuged at 1000 × *g* for 5 minutes. The supernatant was further centrifuged at 100,000 × g for 1 hour at 4 °C to obtain the membrane enriched fraction. The pellet was suspended in Kreb’s buffer (100 mM NaCl, 5 mM KCl, 2 mM CaCl2, 1.2 mM MgSO4, 1.0 mM K2HPO4, 25 mM NaHCO3, 20 mM Na-HEPES, 0.2% glucose, pH 7.4) with PI. The membrane fraction was incubated with lucigenin (5 μM, Invitrogen) at room temperature for 15 min. Then 100 μM of NADPH (Sigma-Aldrich) was added and the chemiluminescence intensity was read with luminometer every 1 minute, up to 10 counts. The data were adjusted to the protein concentration.

### Malondialdehyde (MDA) assay

Lipid peroxidation was measured by MDA assay (Abcam, Cambridge, MA) following the manufacturer’s instructions. Briefly, liver tissues were homogenized on ice in MDA lysis buffer followed by centrifugation at 13,000 × *g* for 10 minutes. The supernatant was incubated with TBA solution at 95 °C. The light absorbance was measured at 532 nm. The values were normalized to the tissue weight.

### RNA extraction and reverse transcription and quantitative real-time polymerase chain reaction

Total RNA was extracted from liver tissues or cells using the RNeasy kit (Qiagen, Germantown, MD). The RNA from human FFPE livers was extracted with a kit from Life Technologies following the instruction. One μg of RNA was reverse-transcribed (iScriptTM cDNA synthesis kit, Bio-Rad, Hercules, CA) and RT-qPCR was performed on the 7900 HT system (Applied Biosystems, Foster City, CA) using Power SYBR Green PCR Master Mix (Applied Biosystems). The absolute amount of target genes was calculated based on the standard curve generated from the templates, and adjusted to the housekeeping gene. The primer sequences used are listed in Suppl. Table 1.

### Luciferase reporter assay

Using the GeneJuice^®^ transfection reagent (MilliporeSigma, Temecula, CA) and following the manufacturer’s protocol, the human stellate cells LX-2 were transfected with the human NOX4 promoter-luciferase reporter construct. The cells were then treated with 100 μM acetaldehyde (MilliporeSigma). After 24 hours, the cells were collected and the luciferase reporter assay was performed (Promega, Madison, WI) following the manufacturer’s instructions. Chemiluminescence for each sample was normalized to the protein content.

### Messenger RNA half-life measurement

LX-2 cells were transduced with either adeno-NOX4 or control virus (adeno-CMV) (Applied Biological Materials Inc. Richmond, BC). To knock down HuR, LX-2 cells were transfected with either siHuR or control siRNA (Thermo Fisher, Waltham, MA) using RiboJuice transfection reagent (MilliporeSigma) following the instruction. The cells then were treated with 2.0 μg/ml actinomycin D (MP Biomedicals, Solon, OH) at the indicated time points. Total RNA was extracted with the TRIzol reagent (Life Technologies) following the manufacturer’s instructions. The amount of CCR2 and CCL2 mRNA was measured by RT-qPCR. mRNA half-life was calculated as T1/2 = Ln(2)/λ.

### Western blotting

LX-2 cells transduced with adeno-NOX4 or adeno-CMV were serum starved for 16 hr. Cytoplasmic proteins were collected using an ER Isolation kit (MilliporeSigma). Fifty micrograms of cytoplasmic proteins were subjected to 10% SDS-polyacrylamide gel electrophoresis (SDS-PAGE) and transferred onto polyvinylidine difluoride (PVDF) membranes. The membranes were blocked and incubated with anti- HuR (Santa Cruz Biotechnology, Santa Cruz, CA) and anti-phospho-HuR (Ser221) (MilliporeSigma), followed by horseradish peroxidase-conjugated secondary antibodies (Santa Cruz Biotechnology). Detection was performed by chemiluminescence method.

### Macrophage migration assay

WT and NOX4KO HSC were isolated and plated in a 24-well plate from a cell migration assay kit (Cell Biolabs, Inc. San Diego, CA). The cells were transfected with a CCR2 expression plasmid DNA (GeneCopoeia, Rockville, MD) using GeneJuice^®^ transfection reagent, followed by transduction with adeno-CCL2 (Vector Biosystems Inc. Malvern, PA). Control plasmid and virus were used in parallel. Twenty four hours later, HSC were washed and macrophages from WT mice were seeded on the upper chambers and co-cultured with HSC. After 12 hours of co-culture, migration was assessed following manufacturer’s instruction. In brief, the non-migrating cells from the upper side of chambers were removed and the cells migrated to the other side were labeled with a fluorescent dye. The cells were then lysed and the fluorescence intensity was measured at 480 nm/520 nm.

### Statistical analysis

All data represent at least three independent experiments and express as the mean ± standard error of the mean (SEM). ANOVA and two-tailed student’s *t*-test were used to analyze the significance of the data. A value of p < 0.05 was considered significant.

## Additional Information

**How to cite this article:** Sasaki, Y. *et al*. NOX4 Regulates CCR2 and CCL2 mRNA Stability in Alcoholic Liver Disease. *Sci. Rep.* **7**, 46144; doi: 10.1038/srep46144 (2017).

**Publisher's note:** Springer Nature remains neutral with regard to jurisdictional claims in published maps and institutional affiliations.

## Supplementary Material

Supplementary File
