# Supplementary material for: NOX4 Regulates CCR2 and CCL2 mRNA Stability in Alcoholic Liver Disease
Source: Sci Rep. 2017 Apr 6;7:46144. doi: 10.1038/srep46144 (PMC5382722; doi:10.1038/srep46144)
Supplement: Supplementary File [file srep46144-s1.pdf]

Supplementary File

NOX4 Regulates CCR2 and CCL2 mRNA Stability in Alcoholic Liver Disease

Yu Sasaki[1,2], Ali Dehnad[1,2], Sarah Fish[1], Ai Sato[1], Joy Jiang[1], Jijing Tian[1], Kathrin Schröder[3], Ralf Brandes[3], Natalie J Török[1,2]

[1]Division of Gastroenterology and Hepatology, University of California Davis, Sacramento, CA, USA, [2]VA Northern California Health Care System, Mather, CA, USA, and [3]Wolfgang Goethe University, Frankfurt am Main, Germany

**Human NOX4**	Forward: 5’-GATGTTGGGGCTAGGATTGTGTC-3’<br>Reverse: 5’-AAAAGGATAAGGCTGCAGTTGAG-3’
**Human CCR2**	Forward: 5’- GACCAGGAAAGAATGTGAAAGTG-3<br>Reverse: 5’-GCTCTGCCAATTGACTTTCCTT-3
**Human CCL2**	Forward: 5’- GTGTCCCAAAGAAGCTGTGA-3<br>Reverse: 5’-AATCCTGAACCCACTTCTGC-3
**Human B2M**	Forward: 5’-TTCTGGCCTGGAGGCTATC-3’<br>Reverse: 5’-TCAGGAAATTTGACTTTCCATTC-3’
**Human Arbp**	Forward: 5’- GAAACTCTGCATTCTCGCTTC-3’<br>Reverse: 5’- GGTGTAATCCGTCTCCACAG-3’
**Mouse NOX4**	Forward: 5’-TTGCCTGGAAGAACCCAAGT-3’<br>Reverse: 5’-TCCGCACAATAAAGGCACAA-3’
**Mouse CCR2**	Forward: 5’-AGAGGCGAAGGCAACAGTCG-3’<br>Reverse: 5’-GCAGGGCCAATGTCTAGTCC-3’
**Mouse CCL2**	Forward: 5’-CTTCTGGGCCTGCTGTTCA-3’<br>Reverse: 5’-CCAGCCTACTCATTGGGATCA-3’
**Mouse TNFα**	Forward: 5’-TCCCAGGTTCTCTTCAAGGGA-3’<br>Reverse: 5’-GGTGAGGAGCACGTAGTCGG-3’
**Mouse IL-1β**	Forward: 5’-CAACCAACAAGTGATATTCTCCATG-3’<br>Reverse: 5’-GATCCACACTCTCCAGCTGCA-3’
**Mouse IL-6**	Forward: 5’-CCCAATTTCCAATGCTCTC -3’<br>Reverse: 5’-TGAATTGGATGGTCTTGGTC -3’
**Mouse Ly6C**	Forward: 5’-TCTGCCCTGCTGCTGCA -3’<br>Reverse: 5’-TGTGCTGGCCACATGCCTG -3
**Mouse Arbp**	Forward: 5’-CAAAGCTGAAGCAAAGGAAGAG-3’<br>Reverse: 5’-AATTAAGCAGGCTGACTTGGTTG-3’

Table 1: Primer sequences used in the study

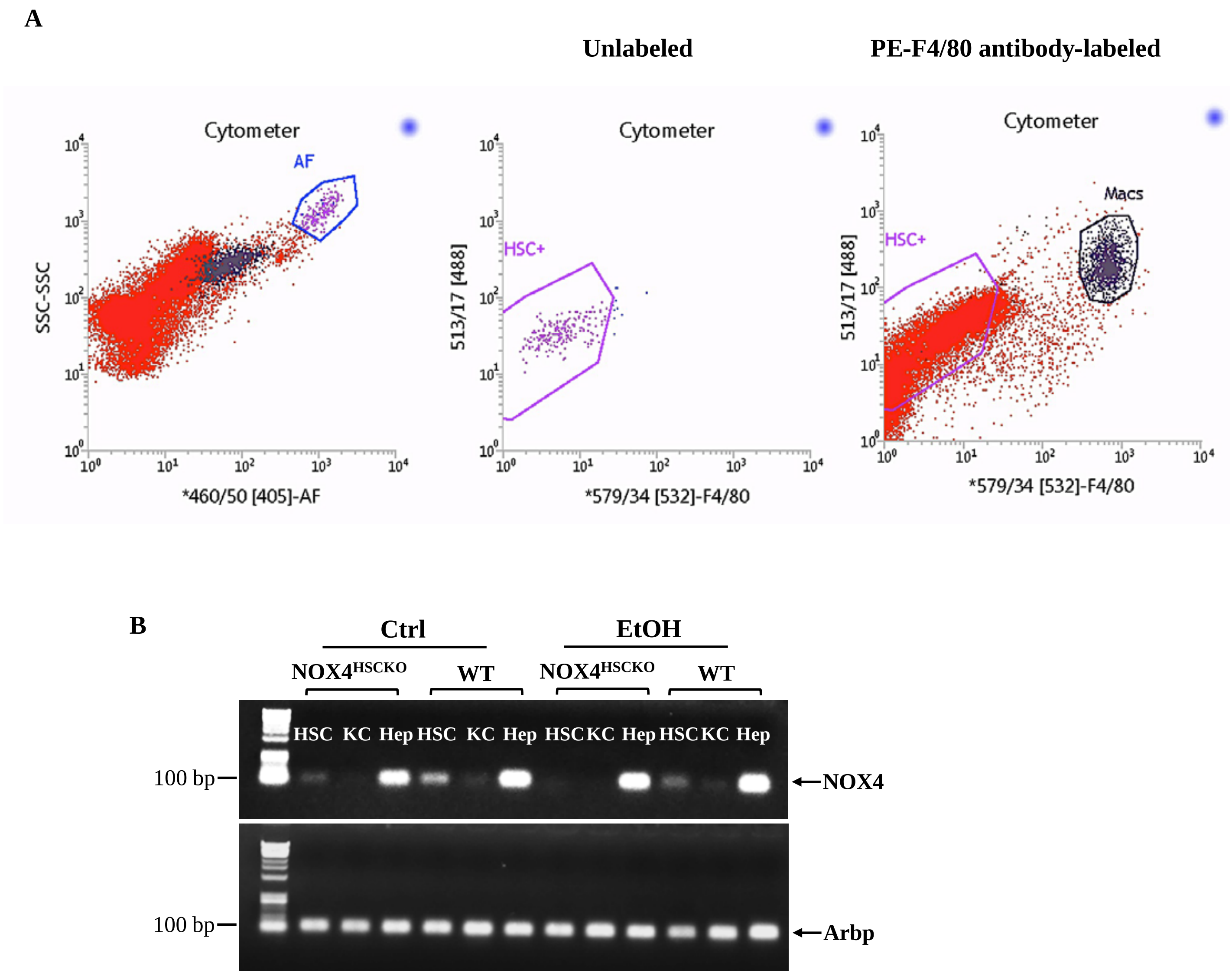

Supplementary Figure 1. Cell isolation, FACS sorting and genotype analysis of HSC, Kupffer cells and hepatocytes isolated from NOX4^{HSCKO} and control (*fl/fl*, denoted as wt) mice.

Hepatocytes and non-parenchymal cells were isolated from WT and NOX4^{HSCKO} mice. (A). HSC from the unlabeled non-parenchymal fraction were first detected with the 460/50 nm filter under the excitation laser 405 nm, based on their autofluorescence (AF, first image). The reference obtained from unlabeled cells was applied to sort HSC (HSC+) and Kupffer cells (Macs). The cells were then subjected to 488nm

laser excitation and detected with PE filter (579/34 nm). After labeling with PE-F4/80 antibody the cells were FACS sorted (Macs).

Hepatocytes, HSC and Kupffer cells were processed for PCR and electrophoresis to detect NOX4 (B). Hepatocytes demonstrated preserved NOX4 expression in NOX4$^{HSCKO\ mice}$, Kupffer cells were not significant sources of NOX4 whereas NOX4 was deleted from HSC from NOX4^{HSCKO} mice.

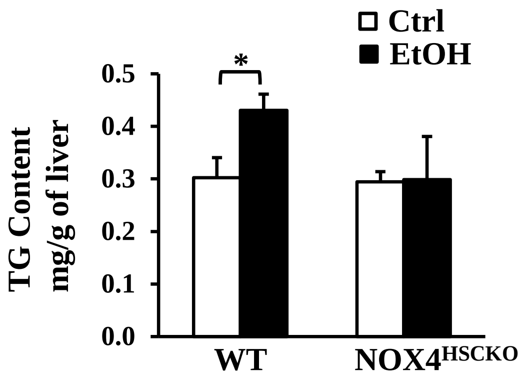

Supplementary Figure 2. Triglyceride content increased in wild type mice on alcohol diet but no increase was noted in NOX4^{HSCKO} mice ($p<0.05$).

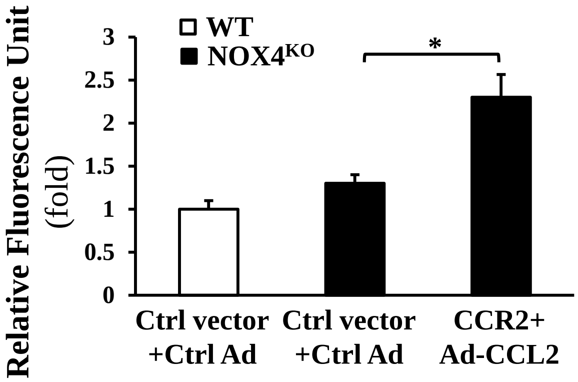

Supplementary Figure 3: CCR2 and CCL2 overexpression in NOX4KO HSC induces macrophage migration in a Transwell system. WT and NOX4KO HSC were isolated and transfected with a CCR2 expression plasmid, followed by transduction with adeno-MCP1 (CCL2). Control plasmid and virus were used in parallel. Twenty four hours later, HSC were washed and macrophages from WT mice were seeded on the upper chambers and co-cultured with HSC. After 12 hours of co-culture, migration was assessed by removing the non-migrating cells from the upper side of chambers and labeling migrated cells with a fluorescent dye. The cells were then lysed and the fluorescence was measured at 480nm/520nm.

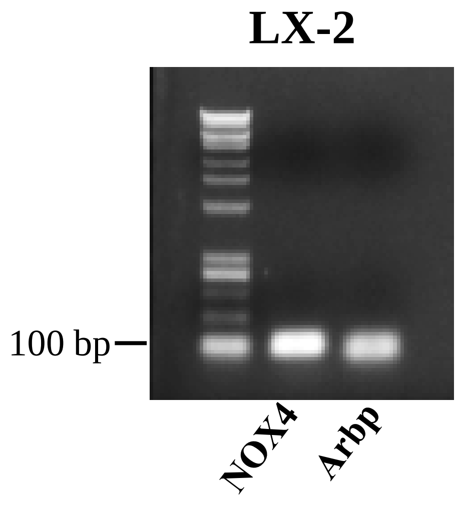

Supplementary Figure 4: LX2 cells express NOX4. Total mRNA was isolated and PCR was performed to demonstrate NOX4 expression in human HSC line.

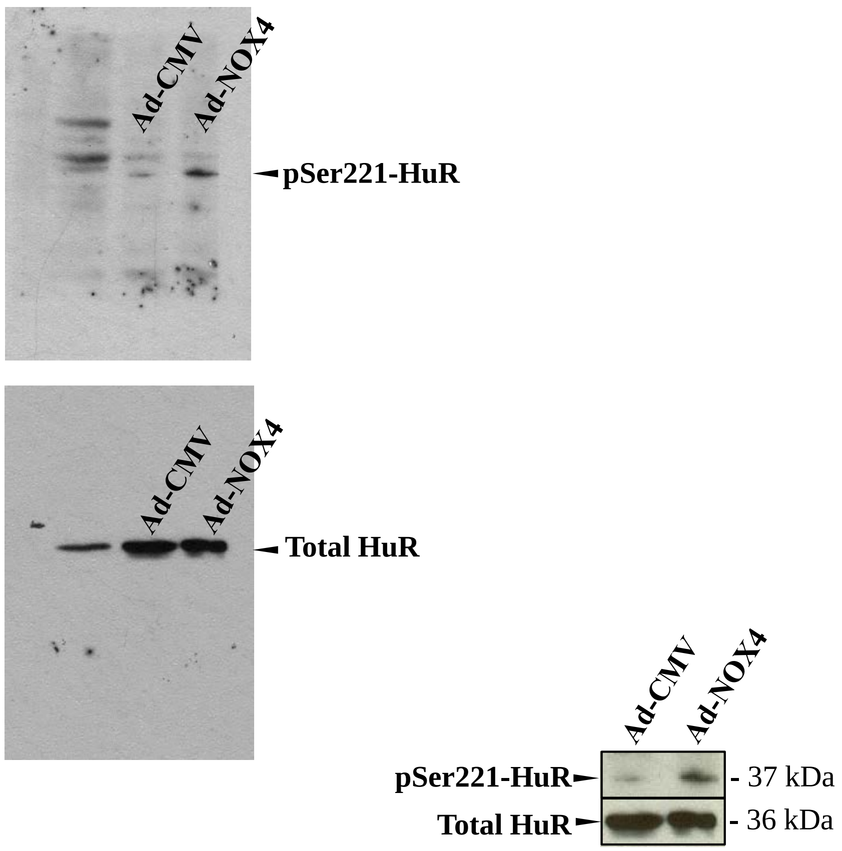

Supplementary Figure 5: Western blot analysis of pSer221 and total HuR showing full-length gel.
